# Volatile Organic Compounds (VOCs) Produced by *Levilactobacillus brevis* WLP672 Fermentation in Defined Media Supplemented with Different Amino Acids

**DOI:** 10.3390/molecules29040753

**Published:** 2024-02-06

**Authors:** Sarathadevi Rajendran, Patrick Silcock, Phil Bremer

**Affiliations:** 1 Department of Food Science, University of Otago, Dunedin 9054, New Zealand; sarathadevi.rajendran@postgrad.otago.ac.nz (S.R.); pat.silcock@otago.ac.nz (P.S.); 2 Department of Agricultural Chemistry, Faculty of Agriculture, University of Jaffna, Kilinochchi 44000, Sri Lanka

**Keywords:** amino acids, defined medium (DM), lactic acid bacteria (LAB), volatile organic compounds (VOCs)

## Abstract

Fermentation by lactic acid bacteria (LAB) is a promising approach to meet the increasing demand for meat or dairy plant-based analogues with realistic flavours. However, a detailed understanding of the impact of the substrate, fermentation conditions, and bacterial strains on the volatile organic compounds (VOCs) produced during fermentation is lacking. As a first step, the current study used a defined medium (DM) supplemented with the amino acids L-leucine (Leu), L-isoleucine (Ile), L-phenylalanine (Phe), L-threonine (Thr), L-methionine (Met), or L-glutamic acid (Glu) separately or combined to determine their impact on the VOCs produced by *Levilactobacillus brevis* WLP672 (LB672). VOCs were measured using headspace solid-phase microextraction (HS-SPME) gas chromatography–mass spectrometry (GC-MS). VOCs associated with the specific amino acids added included: benzaldehyde, phenylethyl alcohol, and benzyl alcohol with added Phe; methanethiol, methional, and dimethyl disulphide with added Met; 3-methyl butanol with added Leu; and 2-methyl butanol with added Ile. This research demonstrated that fermentation by LB672 of a DM supplemented with different amino acids separately or combined resulted in the formation of a range of dairy- and meat-related VOCs and provides information on how plant-based fermentations could be manipulated to generate desirable flavours.

## 1. Introduction

Plant-based foods have gained popularity as consumers choose to reduce their meat and dairy intake due to concerns about their health, the environment, and/or animal welfare [1,2,3,4]. This desire has resulted in an increase in sales of meat or dairy analogues worldwide as consumers seek out alternative forms and flavours of products they are familiar with [5,6,7,8,9,10,11,12]. A challenge with producing plant based-analogues is obtaining realistic meat- or dairy-like flavours.

Flavour is a complex sensory modality that encompasses volatile organic compounds (VOCs) sensed in the nose at the olfactory epithelium retronasally, non-volatile organic compounds sensed on the tongue (taste attributes: sweet, salt, sour, bitter, and umami), and chemesthetic responses (hot, spicy, and pungent) sensed in the oral cavity. Generally, studies on flavour focus on the analysis of VOCs owing to the importance of aroma/odour in overall flavour perception [13,14,15]. VOCs are a low-molecular-weight (<400 Da) compound with a relatively high vapour pressure at room temperature, which means that they can be easily transferred into the gaseous phase [16] and subsequently to olfactory receptors [13,17].

In meat or dairy analogues, the addition of flavour compounds extracted from meat or dairy products is generally not acceptable. In addition, the chemical generation of flavour compounds can be environmentally unfriendly owing to the requirement to use solvents, and such processes lack selectivity, which leads to the formation of unwanted compounds, reduced process efficiency, and increased downstream costs [18]. Although desirable flavours can be extracted directly from plants, this is only economically feasible for a small number of VOCs, as plants contain complex mixtures of VOCs [19]. A promising route for the generation of flavour VOCs that imitate meat or dairy flavour compounds is via the microbial biosynthesis or fermentation of plant material [20]. For example, lactic acid bacteria (LAB) can use plant substrates for energy and nutrition and produce a range of volatile secondary metabolites. In fermented foods, these volatile secondary metabolites are responsible for the production of flavour compounds or flavour precursors [21,22]. The composition of the plant substrates and the LAB strains used have been reported to have the greatest impacts on the resulting fermentation flavours produced [23,24]. However, as the substrate composition of plants can vary widely, understanding how best to generate targeted flavour VOCs through plant-based fermentation is challenging.

LAB are a fastidious microorganism that require a rich cultivation medium for growth, as the majority of them are auxotrophic for a wide range of amino acids and vitamins [25]. Carbohydrates (simple sugars), protein (peptone, yeast extract, beef extract, or whey protein), minerals, vitamins, and buffering agents are common ingredients in LAB cultivation media [26]. A rich cultivation medium, however, is not suitable for determining the role of substrates on VOC formation by LAB fermentation owing to the difficulty in determining which substrates and metabolic pathways are responsible for the VOCs detected. To reduce the complexity in the system, a defined medium with only sufficient nutrients to support LAB growth can be used [27,28,29,30,31,32].

Amino acids are important not only for the growth of LAB but also for the production of flavour compounds. Most amino acids do not have a direct impact on flavour, but they do contribute indirectly because they are precursors to key flavour compounds [33]. Enzymes found in the LAB, such as deaminases, decarboxylases, transaminases (aminotransferases), and lyases, can convert amino acids in different ways. Amino acid transamination is the primary initiator of the conversion of amino acids to flavour compounds. Transamination of amino acids results in α-keto acid formation, which can subsequently be decarboxylated into aldehydes, which in turn can be dehydrogenated into alcohols or carboxylic acids by alcohol dehydrogenases and aldehyde dehydrogenases, respectively [34,35,36].

For LAB strains to be used in commercial production of plant-based flavours, the bacteria need to be readily available, food grade, and not very fastidious in terms of nutrient requirements or growth temperatures. An initial trial with *Levilactobacillus brevis* WLP672 (*Lev. brevis* WLP672) and *Lactobacillus delbrueckii* WLP677 strains identified the LAB brewing strain *Lev. brevis* WLP672 as a good candidate for use in fermentation trials. *Lev. brevis* WLP672 is an obligatory heterofermentative LAB that is used in the production of a wide range of fermented products worldwide. The bacterium uses the phosphoketolase pathway (PK) to ferment hexoses to produce a mixture of lactic acid, ethanol, acetic acid, CO_2_, and an array of volatile secondary metabolites [37].

To date, most studies on the amino acid-derived VOCs produced by yeasts [38,39,40,41,42], fungi [43], and LAB [44,45] have used complex media (natural or synthetic), with only a few studies using LAB in the defined medium [33,46]. This is the first study to use headspace solid-phase microextraction (HS-SPME) gas chromatography–mass spectrometry (GC-MS) to provide a comprehensive analysis of VOCs produced by *Lev. brevis* WLP672 in response to the addition of single or combined amino acids in a defined medium.

## 2. Results and Discussion

### 2.1. Development of Defined Medium

Based on past literature [27,47,48,49,50,51,52] and a series of trials, a defined medium (DM) was developed (Table 1), which supported the growth of *Lev. brevis* WLP672 (thereafter referred to as LB672). In addition to the other components in the DM, growth of LB672 did not occur in the absence of sodium acetate, as previously reported [48]. Sodium acetate is postulated to stimulate the growth of several LAB species, owing to it being both a buffering agent and an energy source [47,48]. Sodium acetate concentrations of 0.1 and 1.2% were the lowest and highest concentrations previously used in nutrient-rich media designed to support the growth of LAB [47,49]. In the current study, sodium acetate stimulated the growth of LB672 in the DM when glucose was present, but it did not enable growth (function as an energy source) when glucose was absent. LB672 also did not grow in the DM in the absence of peptone (enzymatic protein digest, Bacto peptone), even in the presence of individual amino acid. *Lev. brevis* strains have previously been reported to lack genes for amino acid biosynthesis [53,54]. Bacto peptone was used as its composition was defined [55,56]. Further, LB672 did not grow in the absence of the added vitamins or minerals.

To investigate the role of amino acid supplementation on the VOCs produced by LB672 during fermentation, the DM was supplemented with amino acids, either separately or in combination (Table 1). Note that amino acid supplementation in the DM resulted in an amino acid concentration 4 to 30 times higher than the amino acid concentration in the DM (from the peptone) [55,56]. Almost all LAB require glutamic acid (Glu) because of their inability to synthesise its precursor α-ketoglutarate de novo, owing to their lack of a complete TCA cycle [25]. This means aminotransferases, which initiate transamination reactions of amino acid, utilise Glu as the donor substrate of the amino groups and convert them into α-ketoglutarate [36]. To ensure that sufficient Glu was present to enable transamination reactions, Glu was added to all media except the original DM (DM0.1 and DM1.2, Table 1).

### 2.2. Physiochemical Properties

The pH of the medium impacts on the growth of bacteria, and during LAB fermentation, the pH of the medium can decrease owing to the production of lactic acid [57]. In the current study, pH values decreased over the 16 days of fermentation (Table 2) from 5.2 to 4.6 in media containing 1.2% acetate and from 6.6 to 6.1 in media containing 0.1% acetate. During fermentation, the turbidity (OD_600_) of the media increased over time (Table 2) by about 0.5 to 0.7 units in media containing 1.2% acetate and in the range of 0.2 to 0.4 in media containing 0.1% acetate. It is obvious that there were differences obtained in OD_600_ between the two acetate concentrations used in the DM. However, due to the variation in the initial pH of the DM, it is not possible to confirm the impact of acetate on the growth. Further, after 16 days of fermentation in either DM0.1 or DM1.2 (Table 2), OD_600_ values were higher in DM containing 0.1 and 1.2% acetate compared to in DM supplemented with amino acids. The reason for the decreased LB672 growth in the presence of amino acids is unclear. However, as the addition of amino acids decreased the initial pH of the medium, it is speculated that this could be one of the reasons for the decreased growth (lower final OD_600_ values).

### 2.3. Volatile Organic Compounds (VOCs) after Fermentation

**Figure 1:**
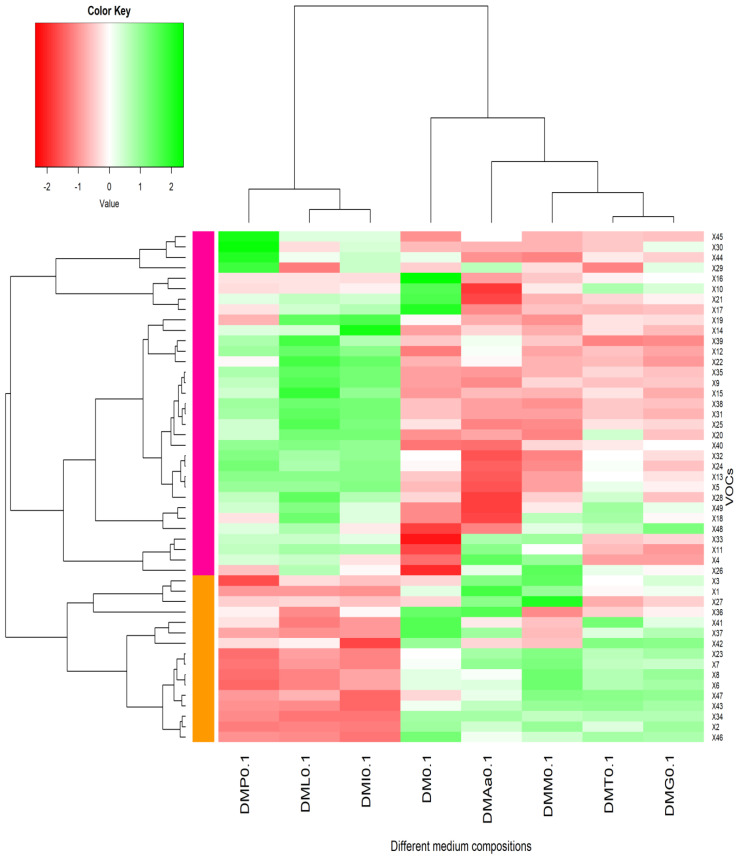
Heatmap visualisation and hierarchical clustering analysis of VOCs produced by LB672 based on the log 2 transformed average peak area of each VOC. Fermentation was carried out in the defined medium (DM) with 0.1% acetate added and supplemented with different amino acids separately or in combination (DMP: Phe added, DML: Leu added, DMI: Ile added, DM: defined medium, DMAa: Aamix added, DMM: Met added, DMT: Thr added, and DMG: Glu added). The green colour represents a higher abundance, whereas the red colour indicates a lower abundance. The VOCs represented in the heatmap are numbered according to the peak numbers (Table S1).

**Figure 2:**
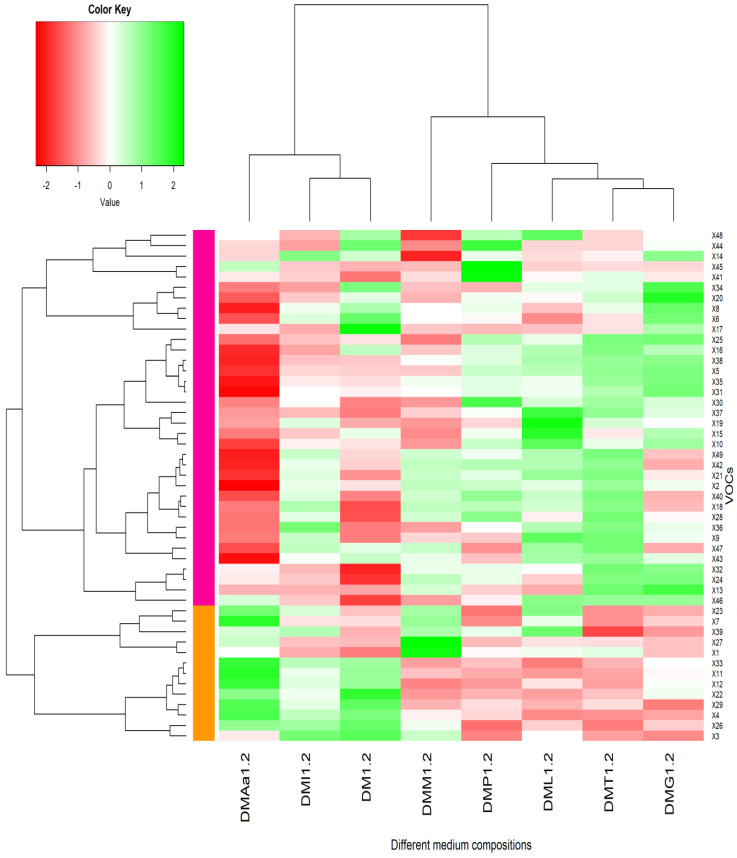
Heatmap visualisation and hierarchical clustering analysis of VOCs produced by LB672 based on the log 2 transformed average peak area of each VOC. Fermentation was carried out in the defined medium (DM) with 1.2% acetate added and supplemented with different amino acids separately or in combination (DMAa: Aamix added, DMI: Ile added, DM: defined medium, DMM: Met added, DMP: Phe added, DML: Leu added, DMT: Thr added, and DMG: Glu added). The green colour represents a higher abundance, whereas the red colour indicates a lower abundance. The VOCs represented in the heatmap are numbered according to the peak numbers (Table S2).

A total of 49 VOCs were detected after 16 days of LB672 fermentation, which were attributed to supplementation of the DM with different amino acids separately or combined. The VOCs detected included alcohols (19 compounds), acids (9), esters (6), sulphur compounds (5), ketones (4), aldehydes (1), and unknown compounds (5) (Table 3). In order to distinguish differences across different medium compositions in the relative abundance of the VOCs detected, hierarchical clustering analysis and heatmap visualisation were carried out (Figure 1 and Figure 2). In the dendrogram, VOCs on nearby branches tend to show positive correlations, whereas distant branches tend to show negative correlations.

In heatmap 1 (Figure 1), VOCs detected after LB672 fermentation in DM containing 0.1% acetate were primarily grouped (column-wise) into two clusters based on the different medium compositions used: cluster 1—phenylalanine (Phe) added (DMP0.1), leucine (Leu) added (DML0.1), and isoleucine (Ile) added (DMI0.1), and cluster 2—DM (DM0.1), amino acid mixture (Aamix) added (DMAa0.1), methionine (Met) added (DMM0.1), threonine (Thr) added (DMT0.1), and Glu only added (DMG0.1). The alcohols (2-methyl propanol, 2-pentanol, 1-hexanol, 1-octanol, 2-octanol, 1-nonanol, 2-nonanol, 1-decanol, and citronellol), Ile/Leu-derived alcohols (2-methyl butanol, and 3-methyl butanol), and Phe-derived compound (phenylethyl alcohol) were present in high proportions in the cluster of Phe, Leu, and Ile, where acids (butanoic acid, hexanoic acid, heptanoic acid, and octanoic acid), Ile/Leu-derived acids (2-methyl butanoic acid, and 3-methyl butanoic acid), and Met-derived compounds (methanethiol, methional, dimethyl disulphide, and dimethyl trisulphide) were in low proportions. In contrast, the alcohols (2-pentanol, 4-methyl-2-heptanol, 1-octanol, 2-octanol, 1-nonanol, 1-decanol, and citronellol) were present in low proportions in the cluster of DM, Aamix, Met, Thr, and Glu, where Met-derived compounds (dimethyl disulphide, and dimethyl trisulphide) and acids (butanoic acid, hexanoic acid, and heptanoic acid) were in high proportions. Further, VOCs were row-wise clustered, mainly into two clusters (Figure 1 and Table S1); cluster 1 (pink) contains higher proportions of alcohols, ketones, Phe-derived compounds, and Leu/Ile-derived alcohols, while cluster 2 (orange) is characterized by acids, Met-derived compounds and Leu/Ile-derived acids.

In DM containing 1.2% acetate, VOCs formed two main clusters (column-wise), as shown by heatmap 2 (Figure 2): an Aamix (DMAa1.2), Ile (DMI1.2), and DM (DM1.2) cluster and a Met (DMM1.2), Phe (DMP1.2), Leu (DML1.2), Thr (DMT1.2), and Glu (DMG1.2) cluster. The alcohols (2-methyl propanol, 1-octanol, 2-octanol, 1-nonanol, 1-decanol) and ketones (2-heptanone, 2-nonanone, and 2-undecanone) were present in low proportions in the cluster of Aamix, Ile, and DM. In the subcluster of Ile and DM, Met-derived compounds (methanethiol, methional, and dimethyl disulphide) and Phe-derived compounds (phenylethyl alcohol, and 2-phenylethyl acetate) were in low proportions. As the Aamix medium contained all of the amino acids, the possibility of forming high proportions of all amino acid-derived VOCs was increased. In contrast to this cluster, the alcohols (2-methyl propanol, 1-octanol, 2-octanol, 1-nonanol, and 1-decanol) were present in high proportions in the subcluster of Phe, Thr, Leu, and Glu. Further, VOCs were row-wise clustered into two clusters (Figure 2 and Table S2); cluster 1 (pink) is characterized by higher proportions of acids, alcohols, ketones, esters, and Leu/Ile/Phe-derived compounds, while cluster 2 (orange) is presented by higher proportions of Met-derived compounds.

Since the heat map analysis highlighted differences between the VOC profile across different medium compositions, the specific amino acid-derived VOCs produced by LB672 across different medium compositions are discussed in the following sections separately.

### 2.4. Phe-Derived VOCs

Phe, an aromatic amino acid, can be converted by LAB into phenyl pyruvate via the action of aromatic aminotransferases (ArAT) in the presence of α-ketoglutarate. The ArAT is active on all aromatic amino acids (tryptophan (Try), Phe, and tyrosine (Tyr)) and also on Leu and Met [36]. Further, phenyl pyruvate can subsequently be converted into benzeneacetaldehyde (phenylacetaldehyde), phenyl acetic acid, phenylethyl alcohol (phenyl ethanol), benzyl alcohol (phenyl methanol), and benzaldehyde by LAB via enzymatic, non-enzymatic, and unknown mechanisms [36,58].

**Figure 3:**
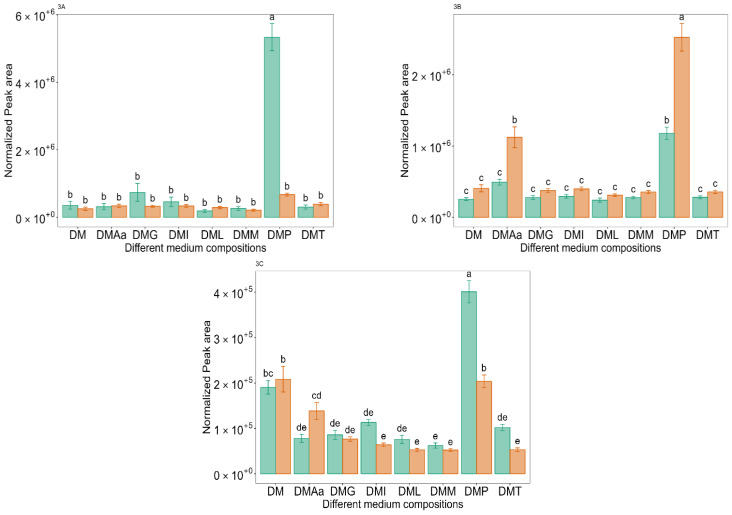
Normalized peak area of benzaldehyde (**3A**), phenylethyl alcohol (**3B**), and benzyl alcohol (**3C**) across different medium compositions (see Table 1) at 0.1% 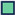 and 1.2% 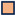 acetate. Values are presented as mean ± standard error (*n* = 6). Different letters represent significant difference between the different medium compositions according to Tukey’s test at *p* < 0.05.

Benzaldehyde, which has characteristic almond and burnt sugar notes [57], is produced chemically (non-enzymatic reactions) from phenyl pyruvate in the presence of oxygen and manganese [34,58]. DM with added Phe and 0.1% acetate had the highest benzaldehyde peak area (Figure 3A, medium DMP). Benzaldehyde was detected in all media possibly due to the presence of peptone, which is speculated to be the reason why many of the compounds discussed in the following sections were also detected in many of the ferments at low levels.

Phenylethyl alcohol, which has a characteristic floral note [59], can be produced from phenyl pyruvate, which is generated by transamination of Phe. Phenylpyruvate is then decarboxylated to phenylacetaldehyde (benzeneacetaldehyde) via the action of decarboxylase. Further, it can be dehydrogenated to phenylethyl alcohol via the action of the enzyme alcohol dehydrogenase (AlcDH) [36]. The highest peak area for phenylethyl alcohol was obtained in media with added Phe, where a significantly (*p* < 0.05) higher peak area was obtained with 1.2% acetate compared to 0.1% acetate (Figure 3B, medium DMP). Phe addition has previously been reported to increase phenylethyl alcohol production during the fermentation of lychee wine using *Saccharomyces cerevisiae* var. *cerevisiae* MERIT.ferm [38], in synthetic grape must fermentation by commercial wine yeast strains [39], in papaya juice fermented by *Williopsis saturnus* var. *mrakii* [41], and in synthetic grape juice fermented by *Saccharomyces cerevisiae* var. *bayanus* [42]. The addition of an amino acid mixture (valine (Val), Leu, Ile, and Phe) to soy (tofu) whey has also been reported to increase the amount of phenylethyl alcohol following *Torulaspora delbrueckii* Biodiva fermentation [40].

Benzyl alcohol, which imparts floral notes, can be produced from benzaldehyde via oxidative and non-oxidative pathways [60]. It was detected at the highest peak area in media supplemented with Phe (Figure 3C, medium DMP), where a significantly (*p* < 0.05) higher amount was obtained with 0.1% acetate compared to 1.2% acetate.

A genomic study by Liu et al. [53] reported that *Lev. Brevis* ATCC 367 lacked the gene *araT* encoding ArAT (putative) enzyme, which initiates the transamination reaction that converts Phe into phenyl pyruvate. However, in the current study, LB672 produced benzaldehyde, phenylethyl alcohol, and benzyl alcohol presumably via the intermediate phenyl pyruvate. Yvon and Rijnen [61] reported that the presence of aspartate aminotransferase (AspAT) in *Brevibacterium linens* is responsible for aspartate (Asp) transamination and is also active on aromatic amino acids. The gene *aspAT* encoding for AspAT enzyme has been detected in several *Lev. Brevis* strains, including *Lev. Brevis* ATCC 367 [53] and *Lev. Brevis* CGMCC 1306 [62]. Hence, it seems more likely that, in the current study, AspAT in LB672 carried out the transamination reaction of Phe rather than the ArAT enzyme.

### 2.5. Met-Derived VOCs

The catabolism of Met, a sulphur-containing amino acid, is initiated by a transamination step involving ArAT or the branched-chain aminotransferase (BcAT) in the presence of α-ketoglutarate, yielding 4-methylthio-2-ketobutyric acid (KMBA). KMBA can be biochemically converted via a decarboxylation reaction into methional and subsequently converted into methanethiol and α-ketobutyrate via an unknown pathway. KMBA can also be directly converted into 2-hydroxyl-4-methylthiobutyric acid and methanethiol via dehydrogenation. Further, demethiolation of Met produces methanethiol, α-ketobutyrate, and ammonia via two pyridoxal phosphate-dependent lyases (cystathionine β-lyase (CBL), cystathionine γ-lyase (CGL)). Lastly, KMBA can be chemically converted into methanethiol [34,36,58,63]. Methanethiol produced through these pathways can be further converted into dimethyl sulphide, dimethyl disulphide, and dimethyl trisulphide by auto-oxidation [36,63]. In addition, methanethiol can also react with carboxylic acids to produce thioesters [58]. The α-ketobutyrate formed from the Met catabolism can be converted into propanoic acid and yields ATP via substrate-level phosphorylation [63].

**Figure 4:**
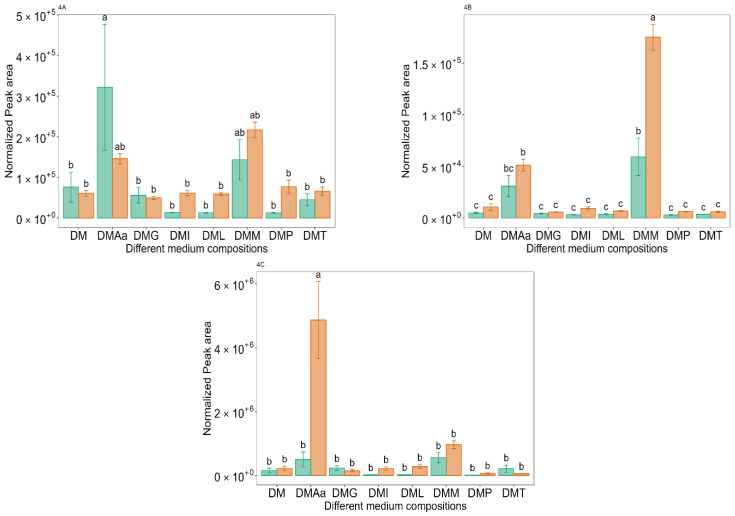
Normalized peak area of methanethiol (**4A**), methional (**4B**), and dimethyl disulphide (**4C**) across different medium compositions (see Table 1) at 0.1% 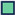 and 1.2% 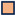 acetate. Values are presented as mean ± standard error (*n* = 6). Different letters represent significant difference between the different medium compositions according to Tukey’s test at *p* < 0.05.

In the current study, the highest peak area for methanethiol was in DM at 0.1% acetate supplemented with the Aamix (Figure 4A, medium DMAa), which contained Met along with other amino acids. The highest peak area for methional was in medium supplemented with Met (Figure 4B, medium DMM), where a significantly (*p* < 0.05) higher peak area was obtained for 1.2% acetate compared to 0.1% acetate. For dimethyl disulphide, the highest peak area was observed in the medium with Aamix added (Figure 4C, medium DMAa) at 1.2% acetate, suggesting that it was converted from methanethiol.

According to a study by Liu et al. [53], the genes *cblA* and *cglA*, which encode CBL and CGL lyases, are present in *Lev. Brevis* ATCC 367, and catalyse the demethiolation reaction of Met. Lyases, play an important role in the biosynthesis of sulphur-containing amino acids but not a major role in Met catabolism by LAB [36]. Therefore, aminotransferases ArAT or BcAT, which might be present in LB672 and involved in the transamination reaction, would appear to be the major source of the degradation products. As the peak area for methanethiol was high and the peak area for methional was low, the results suggest that either methional was produced through transamination (either by ArAT or by BcAT) and converted into methanethiol or that methanethiol was produced directly from Met via the activity of CBL/CGL.

### 2.6. Leu/Ile-Derived VOCs

The catabolism of Leu and Ile (branched-chain amino acids) is initiated by an aminotransferase enzyme, BcAT, that catalyses the hydrolysis of Leu and Ile to the α-keto acids 4-methyl-2-oxopentanoic acid (α-ketoisocaproate) and 3-methyl-2-oxopentanoic acid (α-keto-β-methylvalerate), respectively. In many LAB, ArAT is also involved in the transamination of Leu. After the transamination reaction, α-keto acids undergo the following biochemical reactions: 1. Oxidative decarboxylation of 4-methyl-2-oxopentanoic acid and 3-methyl-2-oxopentanoic acid to 3-methyl butanoic acid and 2-methyl butanoic acid, respectively (enzyme: ketoacid dehydrogenase (KaDH)); 2. Decarboxylation of 4-methyl-2-oxopentanoic acid and 3-methyl-2-oxopentanoic acid to 3-methyl butanal and 2-methyl butanal, respectively (enzyme: keto acid decarboxylase (KdcA)); and 3. Reduction of 4-methyl-2-oxopentanoic acid and 3-methyl-2-oxopentanoic acid to 2-hydroxy-4-methylpentanoic acid and 2-hydroxymethylvalerate, respectively (enzyme: hydroxy acid dehydrogenase (HycDH)). Further, 3-methyl butanal and 2-methyl butanal can be either reduced to alcohols by AlcDH (3-methyl butanol and 2-methyl butanol, respectively) or oxidised to acids by an aldehyde dehydrogenase (AldDH) (3-methyl butanoic acid and 2-methyl butanoic acid, respectively) [34,35,36].

**Figure 5:**
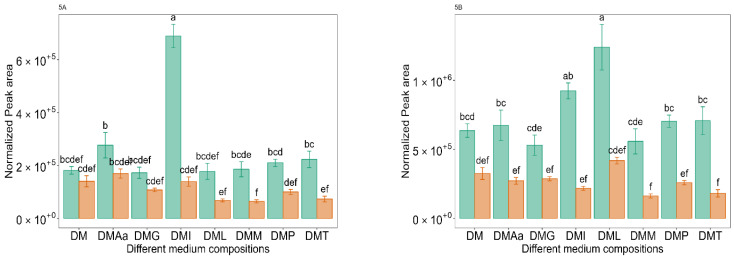
Normalized peak area of 2-methyl butanol (**5A**) and 3-methyl butanol (**5B**) across different medium compositions (see Table 1) at 0.1% 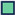 and 1.2% 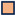 acetate. Values are presented as mean ± standard error (*n* = 6). Different letters represent significant difference between different medium compositions according to Tukey’s test at *p* < 0.05.

In the current study, the highest peak area for 2-methyl butanol was observed in DM supplemented with Ile (Figure 5A, DMI) at 0.1% acetate. Similarly, the highest peak area for 3-methyl butanol was obtained in DM supplemented with Leu (Figure 5B, DML) at 0.1% acetate. The addition of Leu has previously been reported to increase 3-methyl butanol production: in synthetic grape must fermented by commercial wine yeast strains [39], in synthetic cassava medium fermented by *Ceratocystis fimbriata* [43], and in papaya juice fermented by *Williopsis saturnus* var. *mrakii* [41]. The addition of an amino acid mixture (Val, Leu, Ile, and Phe) to soy (tofu) whey has also been reported to increase the peak area of 3-methyl butanol fermented by *Torulaspora delbrueckii* Biodiva [40]. Further, the addition of Ile has also been reported to increase 2-methyl butanol production during the fermentation of papaya juice by *Williopsis saturnus* var. *mrakii* [41].

The gene *bcaT* encodes the BcAT enzyme which initiates the transamination reaction on branched-chain amino acids, and the gene *araT* encodes the ArAT (putative) enzyme that initiates the transamination reaction on Leu, were both absent in a genome study of *Lev. Brevis* ATCC 367 [53]. In the current study, LB672 fermentation resulted in the production of 2- and 3-methyl butanol from Ile and Leu, respectively. This result suggests that LB672 possesses BcAT or ArAT enzymes, which catalysed the transamination of Ile and Leu because 2- and 3-methyl butanol could not have been produced without a transamination step.

**Figure 6:**
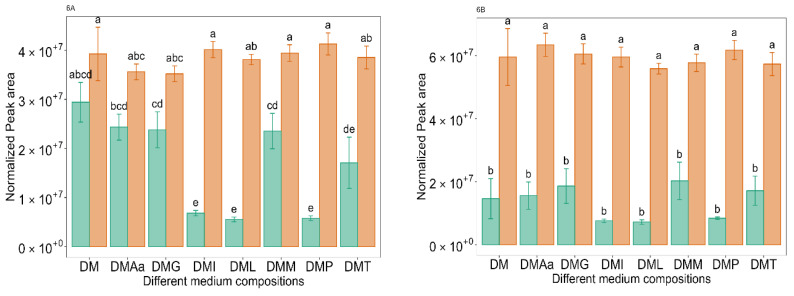
Normalized peak area of ethanol (**6A**) and acetic acid (**6B**) across different medium compositions (see Table 1) at 0.1% 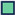 and 1.2% 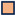 acetate. Values are presented as mean ± standard error (*n* = 6). Different letters represent significant difference between the different medium compositions according to Tukey’s test at *p* < 0.05.

Ethanol, which is a marker compound in fermentation studies, can be produced from sugars via an intermediate acetaldehyde using the PK pathway [21], from the degradation of Thr [64], or from acetate through acetyl-CoA [65]. Overall, the ethanol peak area was higher in DM containing 1.2% acetate compared to 0.1% (Figure 6A). This may have been a result of the acetate being converted into ethanol. Interestingly, in DM supplemented with either Leu, Ile, or Phe at 0.1% acetate, the peak area for ethanol was significantly (*p* < 0.05) lower than in other media with either 0.1 or 1.2% acetate. This finding suggests that LB672 in the presence of added Leu, Ile, or Phe at the lower acetate concentrations is using a pathway other than an ethanol-producing pathway. 

Acetic acid was detected in all media, with its concentration being significantly (*p* < 0.05) higher at 1.2% acetate-containing DM compared to 0.1% acetate-containing DM (Figure 6B). Though *Lev. Brevis* strains have been shown to produce acetic acid in addition to lactic acid [66], owing to the fact that acetate was present in the DM, it is not possible to confirm that LB672 produced additional acetate. Acetic acid, however, is the building block for fatty acids consisting of an even number of carbons [32,67]. In the current study, butanoic, hexanoic, octanoic, and decanoic acids were present in a higher abundance in media containing 1.2% acetate compared to 0.1%. This result demonstrates in a concentration-dependent manner that acetate could be converted into fatty acids with even carbon chain-length numbers.

In the current study, the addition of some individual amino acids (Phe, Met, Leu, and Ile) or the addition of the amino acid mixture (Aamix) had an impact on the relative proportions of VOCs formed by LB672 fermentation. In contrast, no particular VOCs were detected in the medium supplemented with Thr. Thr catabolism by LAB is initiated by threonine aldolase (TA) or serine hydroxymethyltransferase (SHMT), which converts Thr to acetaldehyde and glycine [35,36]. Acetaldehyde was not detected after LB672 fermentation in the current study. As acetaldehyde is an intermediate compound in the ethanol production pathway [64], it was considered that it was most likely not detected rather than not being present. The inability to detect acetaldehyde was due to the fact that the HS-SPME method used was not optimised for its detection—rather, it was designed to detect a broad range of compounds.

The addition of different concentrations of acetate (0.1, or 1.2%) in the DM significantly influenced the relative abundance of VOCs generated after LB672 fermentation. The mechanism(s) underpinning this observation were, however, unclear, as acetate’s role in LAB growth and VOC generation is complicated. In addition to serving as a buffering agent, acetate has also been reported to demonstrate growth-stimulating activities for several LAB [47], and omitting acetate from the media can result in diminished growth [48]. Acetate can also serve as a precursor to a number of lipids/fatty acids [32,68] and ethanol [65]. 

The presence of aminotransferases such as BcAT, ArAT, and AspAT and other enzymes involved in amino acid catabolism, including KdcA, AlcDH, AldDH, and CBL/CGL, in LB672 is likely in the current study because of the production of VOCs derived from branched-chain, aromatic, or sulphur (Met) amino acids. This could be confirmed by applying a genomic analysis of LB672.

In the current study, the VOCs produced after 16 days of fermentation were analysed using GC-MS. Although GC-MS is a commonly used reference method for VOC analysis, it cannot be used to track changes in VOCs in real time. However, over time, the concentration of VOC produced can fluctuate dramatically. It is therefore possible that compounds were produced but were not detected in the analysis performed at the end of the fermentation (16 days). Proton transfer reaction-time of flight-mass spectrometry (PTR-ToF-MS) is a quick, direct, non-invasive, and highly sensitive (parts per trillion by volume) online method that can be used to monitor the production of VOCs [69]. Such an approach would be useful in determining how VOCs change over time in the DM supplemented with different amino acids.

Among the fermentation-derived VOCs detected in the present study, dimethyl trisulphide, methional, 2-nonanone, 2-undecanone, 1-hexanol, and 1-octanol were characteristic odour-active VOCs in cooked meat [70]; acetic acid, 3-methyl butanoic acid, methional, dimethyl trisulphide, and hexanoic acid were odour-active VOCs in dairy yoghurt [71]; and acetic acid, butanoic acid, 3-methyl butanoic acid, 3-methyl butanol, methional, methanethiol, dimethyl disulphide, dimethyl trisulphide, 2-phenylethyl acetate, benzaldehyde, ethyl hexanoate, 2-heptanone, 2-nonanone, and 2-undecanone were odour-active VOCs in dairy cheese [61,72]. Hence, the current study has reinforced the critical role that substrate composition plays in VOC production, and the knowledge gained will help researchers to develop plant-based fermentations designed to generate specific VOCs that, when added to a product, will contribute to meat or dairy flavours. For example, using a plant-based substrate high in methionine or cysteine is likely to result in the production of higher concentrations of sulphur-derived VOCs such as methional, methanethiol, methionol, dimethyl sulphide, dimethyl disulphide, and dimethyl trisulphide, which are important contributors to meat or dairy flavours.

## 3. Materials and Methods

### 3.1. LAB Strain

The LB672 culture was obtained from White Labs, USA. A stock culture was maintained at 4 °C until use. For working cultures, LB672 was initially cultivated in de Man, Rogosa, and Sharpe (MRS) broth at 25 °C for 3 days in sealed containers using anaerobic packs (Mitsubishi Gas Chemical (MGC) Company, Tokyo, Japan). An aliquot of the resulting culture was streaked onto MRS agar plates, which were incubated at 25 °C in sealed containers containing MGC anaerobic packs for 3 days or until large colonies were visible on the plates. An inoculating suspension was created by picking colonies from the streak plate and adding them to 10 mL of MRS broth, which was incubated at 25 °C for 3 days in sealed containers using MGC anaerobic packs. To prevent carryover of medium components to fermentation experiments, the cells were added to an Eppendorf tube (1 mL) and centrifugated (5000× *g* for 5 min at 20 °C) using a microcentrifuge (IEC Micromax, Milford, MA, USA). Cells were then washed twice with sterilised phosphate-buffered saline (PBS) (100 mL; 0.8 g NaCl, 0.02 g KCl, 0.144 g Na_2_HPO_4_, and 0.0245 g KH_2_PO_4_, pH of 7.4) and then resuspended to a final concentration of 1 ×10^9^ CFU/mL. The resulting cell suspension was used for the fermentation studies.

### 3.2. Medium Compositions

The base for the DM contained D-glucose (20 g/L); peptone (enzymatic protein digest, Bacto peptone) (5 g/L), sodium acetate (either 1 or 12 g/L), mineral salts (MgSO_4_·7H_2_O (0.2 g/L), NaCl (0.01 g/L), FeSO_4_.7H_2_O (0.01 g/L), and MnSO_4_.5H_2_O (0.04 g/L)), and vitamins (calcium pantothenate (B5) (0.4 mg/L), nicotinic acid (B3) (0.2 mg/L), riboflavin (B2) (0.4 mg/L), and thiamine HCl (B1) (0.2 mg/L)). The DM was supplemented with amino acids L-leucine (Leu) (media DML0.1 and DML1.2), L-isoleucine (Ile) (media DMI0.1 and DMI1.2), L-phenylalanine (Phe) (media DMP0.1 and DMP1.2), L-threonine (Thr) (media DMT0.1 and DMT1.2), L-methionine (Met) (media DMM0.1 and DMM1.2), or L-glutamic acid (Glu) (media DMG0.1 and DMG1.2) separately (2 g/L) or combined (0.4 g/L each) (Aamix) (media DMAa0.1 and DMAa1.2) (Table 1). Glu was added to all media (2 g/L) except the two-base DM without added amino acid (media DM0.1 and DM1.2). The pH of the DM containing 0.1% acetate was adjusted to 6.6 using K_2_HPO_4_ (1M) and a pH meter (pH 213 microprocessor, HANNA, Cluj-Napoca, Romania). All the chemicals used were analytical grade unless otherwise stated. Most components were prepared as concentrated stock solutions and stored at 4 °C until used. The amino acids were dissolved in HCl solution (50 mM). All the stock solutions were prepared using reverse osmosis (RO) water unless otherwise stated. During the media preparation, the glucose and vitamin solutions were filter (nylon membrane: 0.22 µm, BIOFIL, Kowloon, Hong Kong) sterilised, with the other components being sterilised via autoclaving (121 °C, 15 min) (Astell Scientific, Sidcup, UK). In a class II biological safety cabinet (NUAIRE/NU-425-400, Plymouth, MN, USA), the sterilised media components were dispensed into sterile Schott bottles (100 mL) to achieve a final volume of 50 mL.

### 3.3. Fermentation

To confirm their sterility, prepared media were incubated at 25 °C for at least three days prior and checked for an absence of turbidity prior to inoculation. Each medium was inoculated with 100 µL of the LB672 cell suspension (1 × 10^9^ CFU/mL) and incubated at 25 °C for 16 days under anaerobic conditions, as mentioned previously. Uninoculated sterile medium was used as control. All the fermentations were carried out in duplicate. At the end of the fermentation, growth was confirmed by measuring the pH and optical density (OD_600_, Ultrospec 3300 pro, Amersham Biosciences, Amersham, UK) of an aseptically removed sub-sample.

### 3.4. Determination of Volatile Organic Compounds

The volatile organic compounds (VOCs) present at the end of fermentation were measured using headspace–solid phase microextraction (HS-SPME) gas chromatography–mass spectrometry (GC-MS) (Agilent 6890N GC system, Beijing, China; coupled to an Agilent 5975B VL mass spectrometer with triple axis detector, Wilmington, DE, USA). Samples were prepared by centrifuging (2000 rpm, 4 °C for 10 min, Sorvall RT 6000, Wilmington, DE, USA) a sub-sample (35 mL) of the ferment and adding the resulting supernatant (10 mL) to a 20 mL headspace vial (Phenomenex, Auckland, New Zealand) containing 3 g of NaCl along with 20 μL of the internal standard (3-heptanone (Sigma-Aldrich, Saint Louis, MO, USA) (0.008 mg/mL, prepared with methanol)). The vials were quickly capped with a Teflon-faced septa and stored at 4 °C in a refrigeration block (Peltier cooling tray) in the autosampler until analysis. Three analytical replicates were prepared from each supernatant to provide a total of six replicates for each fermentation medium (biological replicates: 2 and analytical replicates: 3). Samples were analysed in a randomised order, blocked by replicates. At the beginning of each replicate and at the end of each replicate, two blank samples consisting solely of RO water were analysed.

Prior to GC-MS analysis, each headspace vial was incubated at 40 °C for 5 min, after which the SPME fibre (divinylbenzene/carboxen/polydimethylsiloxane (DVB/CAR/PDMS), 50/30 µm Stableflex, Supelco, Bellefonte, PA, USA) was inserted through the septum of the vial and exposed to the headspace for 40 min at 40 °C. Upon completion of the extraction procedure, for thermal desorption, the fibre was inserted into the front injector port of the GC (set at 230 °C) for 2 min in splitless mode followed by 3 min in split mode (purge flow: 50 mL/min). Between samples, the SPME fibre was conditioned in the back inlet at 270 °C with a purge flow of 50 mL/min for 2 min. VOCs were separated through a Zebron ZB-Wax capillary column (60 m × 0.32 mm inner diameter × 0.5 μm film thickness: Phenomenex, Torrance, CA, USA) using helium as the carrier gas at a constant flow rate of 1 mL/min. The oven temperature programme was set at 50 °C for 5 min initially, followed by heating at a rate of 5 °C/min to 210 °C, followed by 10 °C/min until 240 °C, and finally held for 5 min, for a total run time of 45 min. Mass ions were measured between 29 and 300 m/z via electron ionisation (EI mode 70 eV). The ion source temperatures of mass ion traps were sustained at 230 °C with a quadrupole temperature of 150 °C.

### 3.5. Data Analysis

#### 3.5.1. GC-MS Data Extraction

The GC-MS raw data files were exported in CDF format and imported into PARADISe (Version V6.0.0.14) software. PARADISe is based on PARAFAC2 modelling, which allowed simultaneous deconvolution of pure mass spectra of peaks and integration of areas of deconvoluted peaks for all samples. Resolved peaks were identified by matching their deconvoluted pure mass spectra and retention index (RI) [73,74] against the NIST spectral library (NIST14, version 2.2, National Institute of Standards and Technology). The compounds’ RI (NIST spectral library) was compared using the calculated RI. A cubic spline interpolation using the n-alkane series (C7–C25), which was run under the same conditions in GC-MS, was used to calculate the RI of each compound [75]. The compounds were further selected based on their R match greater than 800. On a few occasions, compounds with R matches below 800 were chosen because they co-eluted with background or air peaks. If a compound only had a calculated RI or if its identification was not confident, VOCs were considered “unknown”. Finally, the data matrix of the peak area for each compound (including not-identified peaks (unknown)) for each sample replicate was exported from PARADISe.

#### 3.5.2. Statistical Analysis

The data exported from PARADISe were examined and compounds that had a significant difference in peak area between controls and treatments were identified using *t*-tests. Significant differences across treatments were assessed via one-way analysis of variance (ANOVA) with a generalised linear model (significance level at *p* < 0.05) using SPSS (IBM SPSS statistics, version 29.0.0.0 (241)).

Statistical analysis for selected compounds was carried out in R (version R 4.2.1) (R Foundation for Statistical Computing, Vienna, Austria). Heatmaps were developed using “cluster”, “gplots”, “factoextra”, and “tidyverse” external packages as a non-targeted approach to encompass variations between medium compositions and selected VOCs. The peak area average of each VOC was log 2 transformed and used to create heatmaps. The VOCs were clustered using a distant matrix computed with the Pearson correlation coefficient.

One-way ANOVA was performed for each selected VOC across different medium compositions for the targeted approach. The mean separations were calculated using Tukey’s HSD test at *p* < 0.05. Graphs were plotted using the “ggplot2” external package in R.

## 4. Conclusions

The use of a defined medium (DM) helped to understand the impact of amino acid addition on the generation of specific fermentation VOCs. When an Aamix or either Phe, Met, Leu, or Ile were added to the DM, the VOC profile produced was noticeably affected after LB672 fermentation; however, no specific VOCs were detected in Thr-supplemented DM. The results provide a foundation for understanding LB672’s role in amino acid catabolism by indicating which amino acid catabolic enzymes may be present and by highlighting the VOC they produce. To gain a better understanding of how specific VOCs could be produced during LAB fermentation, different amino acid combinations and LAB strains of the same or different species should be studied using an online VOC-tracking method (PTR-ToF-MS).

## Figures and Tables

**Table 1:** Overview of the composition of the different media used.

Media	Glucose	Peptone	Vitamins	Salt	Sodium Acetate	Glu	Leu	Ile	Phe	Thr	Met
DML0.1	2%	0.5%	√	√	0.1%	0.2%	0.2%	-	-	-	-
DMI0.1	2%	0.5%	√	√	0.1%	0.2%	-	0.2%	-	-	-
DMP0.1	2%	0.5%	√	√	0.1%	0.2%	-	-	0.2%	-	-
DMT0.1	2%	0.5%	√	√	0.1%	0.2%	-	-	-	0.2%	-
DMM0.1	2%	0.5%	√	√	0.1%	0.2%	-	-	-	-	0.2%
DMG0.1	2%	0.5%	√	√	0.1%	0.2%	-	-	-	-	-
DM0.1	2%	0.5%	√	√	0.1%	-	-	-	-	-	-
DMAa0.1	2%	0.5%	√	√	0.1%	0.2%	0.04%	0.04%	0.04%	0.04%	0.04%
DML1.2	2%	0.5%	√	√	1.2%	0.2%	0.2%	-	-	-	-
DMI1.2	2%	0.5%	√	√	1.2%	0.2%	-	0.2%	-	-	-
DMP1.2	2%	0.5%	√	√	1.2%	0.2%	-	-	0.2%	-	-
DMT1.2	2%	0.5%	√	√	1.2%	0.2%	-	-	-	0.2%	-
DMM1.2	2%	0.5%	√	√	1.2%	0.2%	-	-	-	-	0.2%
DMG1.2	2%	0.5%	√	√	1.2%	0.2%	-	-	-	-	-
DM1.2	2%	0.5%	√	√	1.2%	-	-	-	-	-	-
DMAa1.2	2%	0.5%	√	√	1.2%	0.2%	0.04%	0.04%	0.04%	0.04%	0.04%

Amino acids: glutamic acid (Glu), leucine (Leu), isoleucine (Ile), phenylalanine (Phe), threonine (Thr), methionine (Met).

**Table 2:** The pH and OD_600_ of samples after 16 days of fermentation by LB672 in different medium compositions.

Media	Initial pH	After 16 Days of Fermentation	
		pH	OD_600_
DML0.1	6.66	6.24	0.269 ^de^
DMI0.1	6.64	6.30	0.271 ^de^
DMP0.1	6.65	6.17	0.223 ^e^
DMT0.1	6.68	6.10	0.297 ^de^
DMM0.1	6.67	6.09	0.249 ^de^
DMG0.1	6.83	6.20	0.286 ^de^
DM0.1	7.85	7.28	0.391 ^cd^
DMAa0.1	6.63	6.45	0.233 ^e^
DML1.2	5.27	4.63	0.511 ^bc^
DMI1.2	5.28	4.64	0.536 ^bc^
DMP1.2	5.29	4.72	0.542 ^bc^
DMT1.2	5.26	4.66	0.517 ^bc^
DMM1.2	5.3	4.66	0.515 ^bc^
DMG1.2	5.4	4.89	0.605 ^ab^
DM1.2	7.01	5.0	0.711 ^a^
DMAa1.2	5.31	4.87	0.492 ^bc^

Results are the mean value of duplicate samples. Values with different superscript lowercase letters (^a–e^) in the column (OD_600_) are significantly different according to Tukey’s test at *p* < 0.05. DML0.1 to DMAa1.2: media supplemented with different amino acids (see Table 1).

**Table 3:** LB672 fermentation VOCs identified in different medium compositions via HS-SPME-GC-MS analysis.

No.	Compound Name	RI (Calc.)	RI (Lit.)	R Match	Identification Method
	Alcohols				
1	Ethanol	930	932	942	MS, RI
2	2-Methyl propanol	1086	1092	827	MS, RI
3	2-Pentanol	1116	1119	881	MS, RI
4	2-Methyl butanol	1199	1208	905	MS, RI
5	3-Methyl butanol (isoamyl alcohol)	1200	1209	962	MS, RI
6	3-Heptanol	1287	1290	959	MS, RI
7	2-Heptanol	1310	1320	953	MS, RI
8	3-Methyl-2-buten-1-ol (prenol)	1312	1320	815	MS, RI
9	1-Hexanol	1343	1355	832	MS, RI
10	4-Methyl-2-heptanol	1349	1372	900	MS, RI
11	2-Octanol	1410	1412	849	MS, RI
12	2-Nonanol	1508	1521	952	MS, RI
13	1-Octanol	1545	1557	912	MS, RI
14	1-Nonanol	1647	1660	883	MS, RI
15	1-Decanol	1751	1760	824	MS, RI
16	Citronellol	1752	1765	801	MS, RI
17	Geraniol	1833	1847	879	MS, RI
18	Benzyl alcohol	1868	1870	924	MS, RI
19	Phenylethyl alcohol	1904	1906	914	MS, RI
	Acids				
20	Acetic acid	1429	1449	946	MS, RI
21	Butanoic acid	1620	1625	905	MS, RI
22	2-Methyl butanoic acid	1659	1662	929	MS, RI
23	3-Methyl butanoic acid (isovaleric acid)	1658	1666	956	MS, RI
24	Hexanoic acid	1836	1846	903	MS, RI
25	Heptanoic acid	1944	1950	864	MS, RI
26	Octanoic acid	2049	2060	920	MS, RI
27	Nonanoic acid	2154	2171	912	MS, RI
28	n-Decanoic acid	2261	2276	801	MS, RI
	Esters				
29	Ethyl acetate	889	888	957	MS, RI
30	Butyl acetate	1069	1074	959	MS, RI
31	3-Methylbutyl acetate	1120	1122	867	MS, RI
32	Ethyl hexanoate	1230	1233	860	MS, RI
33	Ethyl heptanoate *	1331	1331	771	MS, RI
34	2-Phenylethyl acetate	1813	1813	800	MS, RI
	Sulphur compounds				
35	Methanethiol *	690	692	734	MS, RI
36	Dimethyl disulphide	1072	1077	965	MS, RI
37	Dimethyl trisulphide	1386	1377	935	MS, RI
38	Methional	1455	1454	809	MS, RI
39	5-Ethenyl-4-methyl thiazole	1527	1520	917	MS, RI
	Ketones				
40	4-Methyl-4-penten-2-one	1069	1110	804	MS, RI
41	2-Heptanone	1183	1182	921	MS, RI
42	2-Nonanone	1390	1390	954	MS, RI
43	2-Undecanone	1600	1598	939	MS, RI
	Aldehydes				
44	Benzaldehyde	1530	1520	961	MS, RI
	Unknown compounds				
45	Unknown 1	1027	NA		
46	Unknown 2	1139	NA		
47	Unknown 3	1181	NA		
48	Unknown 4	1597	NA		
49	Unknown 5	1803	NA		

Identification method: MS—mass spectrum, RI—retention indices. *: co-eluted with air peaks/background compounds.

## Data Availability

Data will be made available on request.

## Supplementary Materials

The following supporting information can be downloaded at: https://www.mdpi.com/article/10.3390/molecules29040753/s1, Table S1: VOCs detected in 0.1% acetate media; Table S2: VOCs detected in 1.2% acetate media.
