# Supplementary material for: Volatile Organic Compounds (VOCs) Produced by Levilactobacillus brevis WLP672 Fermentation in Defined Media Supplemented with Different Amino Acids
Source: Molecules. 2024 Feb 6;29(4):753. doi: 10.3390/molecules29040753 (PMC10892824; doi:10.3390/molecules29040753)
Supplement: Supplementary file 1 [file molecules-29-00753-s001.zip › molecules-2808583-supplementary.pdf]

Table S1: VOCs detected in 0.1 % acetate media

X45	Phenylethyl Alcohol	
X30	Benzaldehyde	
X44	Benzyl alcohol	
X29	5-Ethenyl-4-methyl thiazole	
X16	Ethyl hexanoate	
X10	Isoamyl acetate	
X21	Ethyl heptanoate	
X17	3-Heptanol	
X19	3-methyl-2-buten-1-ol (Prenol)	
X14	2-Methyl-1-butanol	
X39	Citronellol	
X12	Unknown	
X22	4-Methyl-2-heptanol	
X35	1-Nonanol	
X9	2-Pentanol	
X15	3-Methyl-1-butanol (Isoamyl alcohol)	
X38	1-decanol	Phe-derived,Leu/Ile-derived alcohols, alcohols, ketones
X31	1-Octanol	
X25	2-Octanol	
X20	1-Hexanol	
X40	Unknown	
X32	2-Undecanone	
X24	2-Nonanone	
X13	2-Heptanone	
X5	2-Methyl-1-propanol	
X28	2-Nonanol	
X49	n-Decanoic acid	
X18	2-Heptanol	
X48	Nonanoic acid	
X33	Unknown	
X11	Unknown	
X4	Unknown	
X26	Acetic acid	
X3	Ethanol	
X1	Methanethiol	
X27	Methional	
X36	2-Methyl-1-butanoic acid	
X41	2-Phenylethyl acetate	Met-derived/leu/Ile-derived acids, acids
X37	3-Methyl-1-butanoic acid (Isovaleric acid)	
X42	Geraniol	
X23	Dimethyl trisulfide	
X7	Dimethyl disulfide	

X8	4-Methyl-4-penten-2-one
X6	Butyl acetate
X47	Octanoic acid
X43	Hexanoic acid
X34	Butanoic acid
X2	Ethyl Acetate
X46	Heptanoic acid

Table S2: VOCs detected in 1.2 % acetate media

X48	Nonanoic acid		Phe/Leu/Ile-derived, acids, alcohols, esters, ketones
X44	Benzyl alcohol		
X14	2-Methyl-1-butanol		
X45	Phenylethyl Alcohol		
X41	2-Phenylethyl acetate		
X34	Butanoic acid		
X20	1-Hexanol		
X8	4-Methyl-4-penten-2-one		
X6	Butyl acetate		
X17	3-Heptanol		
X25	2-Octanol		
X16	Ethyl hexanoate		
X38	1-decanol		
X5	2-Methyl-1-propanol		
X35	1-Nonanol		
X31	1-Octanol		
X30	Benzaldehyde		
X37	3-Methyl-1-butanoic acid (Isovaleric acid)		
X19	3-methyl-2-buten-1-ol (Prenol)		
X15	3-Methyl-1-butanol (Isoamyl alcohol)		
X10	Isoamyl acetate		
X49	n-Decanoic acid		
X42	Geraniol		
X21	Ethyl heptanoate		
X2	Ethyl Acetate		
X40	Unknown		
X18	2-Heptanol		
X28	2-Nonanol		
X36	2-Methyl-1-butanoic acid		
X9	2-Pentanol		
X47	Octanoic acid		
X43	Hexanoic acid		
X32	2-Undecanone		
X24	2-Nonanone		
X13	2-Heptanone		
X46	Heptanoic acid		
X23	Dimethyl trisulfide		Met-derived compounds
X7	Dimethyl disulfide		
X39	Citronellol		
X27	Methional		
X1	Methanethiol		
X33	Unknown		
X11	Unknown		
X12	Unknown		

X22	4-Methyl-2-heptanol
X29	5-Ethenyl-4-methyl thiazole
X4	Unknown
X26	Acetic acid
X3	Ethanol
